# Management of multiple primary prostate malignant tumors: case report of synchronous prostatic adenocarcinoma and lymphoma

**DOI:** 10.3389/fonc.2025.1583504

**Published:** 2025-06-12

**Authors:** Weihuang Zhuang, Huiqiang Wu, Zhiyin Cai, Nina Cai, Weihui Liu, Wanyi Liu

**Affiliations:** ^1^ Department of Hematology, The Second Affiliated Hospital, Fujian Medical University, Quanzhou, Fujian, China; ^2^ The Second Affiliated Hospital of Fujian Medical University, Quanzhou, Fujian, China; ^3^ The Second Clinical Medical College, Fujian Medical University, Quanzhou, Fujian, China; ^4^ Department of Urology, The Second Affiliated Hospital of Fujian Medical University, Quanzhou, Fujian, China

**Keywords:** multiple primary prostate cancer, tumor heterogeneity, treatment, case report, CLL - chronic lymphoblastic leukemia, TP53

## Abstract

The occurrence of multiple primary prostate malignant tumors is extremely rare and there is no consensus on its diagnosis and treatment. This article presents an elderly male patient with both acinar adenocarcinoma of the prostate and small lymphocytic lymphoma (SLL) invading the prostate. He was treated with ibrutinib for the lymphoma and goserelin and bicalutamide for the prostate cancer, resulting in control of both conditions. This case highlights the complexity of diagnosing and managing multiple synchronous prostate malignancies. TP53 mutation may play a central role in dual clonal evolution. Multidisciplinary collaboration and individualized treatment plans are essential in managing rare presentations of prostate MPMT.

## Introduction

The term “multiple primary malignant tumors” (MPMT) is used to describe the occurrence of two or more primary malignant tumors in the same or different organs and tissues of the same patient, either simultaneously or sequentially (1). In recent years, the rate of detection of MPMT has increased significantly, owing to two main factors. Firstly, the population is ageing, and secondly, there has been a continuous advancement in tumor screening and diagnostic techniques (2). However, the precise pathogenesis of this condition remains to be elucidated, and its etiology may be multifactorial, involving genetic susceptibility, chronic inflammatory state, abnormal immune function, altered internal environment, and prior receipt of radiotherapy (3). Currently, most MPMT studies have focused on comorbidity between solid tumors, and cases of coexistence between solid tumors and hematological neoplasms are rare, especially within the same organ, resulting in a lack of relevant clinical knowledge and therapeutic experience.

Prostate cancer is among the most prevalent urological malignant neoplasms in elderly males, with incidence and mortality rates that place it second and fifth among male tumors worldwide, respectively (4). The pathological types of malignant tumors of the prostate include epithelial tumors, neuroendocrine tumors, undifferentiated pleomorphic sarcomas, hematological tumors and others (5). Prostate adenocarcinoma is the most prevalent form of the disease, while prostate lymphoma is extremely rare, with an incidence rate of less than 1% (6). Co-occurrence of prostate adenocarcinoma and lymphoma is extremely rare, with complex clinical manifestations, difficult diagnostic and therapeutic decisions, and a lack of systematic research and clinical guidance on the epidemiologic features, diagnostic strategies, and therapeutic pathways of MPMT.

In the present article, we present a case of MPMT with prostate infiltration in combined chronic lymphocytic leukemia/small lymphocytic lymphoma (CLL/SLL) with vesicular adenocarcinoma of the prostate. We have conducted a systematic review of the clinical manifestations, imaging features, pathological findings, and therapeutic process. We have also discussed the possible pathogenic mechanisms, diagnostic points, and individualized treatment strategies in light of the relevant literature. Our aim is to provide guidance for the identification and management of such rare cases. The objective of this study is to furnish a reference for the identification and management of these rare cases.

## Case report

**Figure 1:**
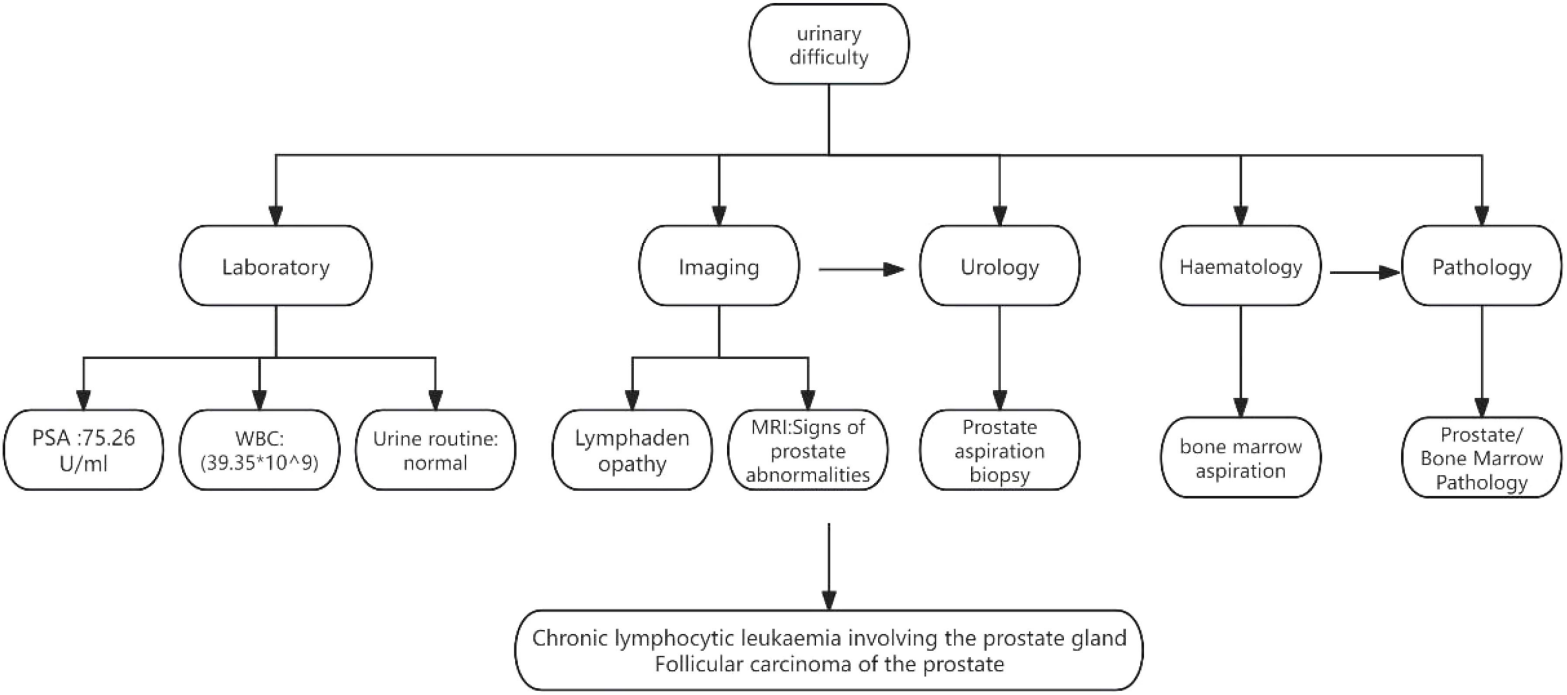
Diagnosis flowchart. PSA, Prostate-Specific Antigen; WBC, White Blood Cell; MRI, Magnetic Resonance Imaging.

**Figure 2:**
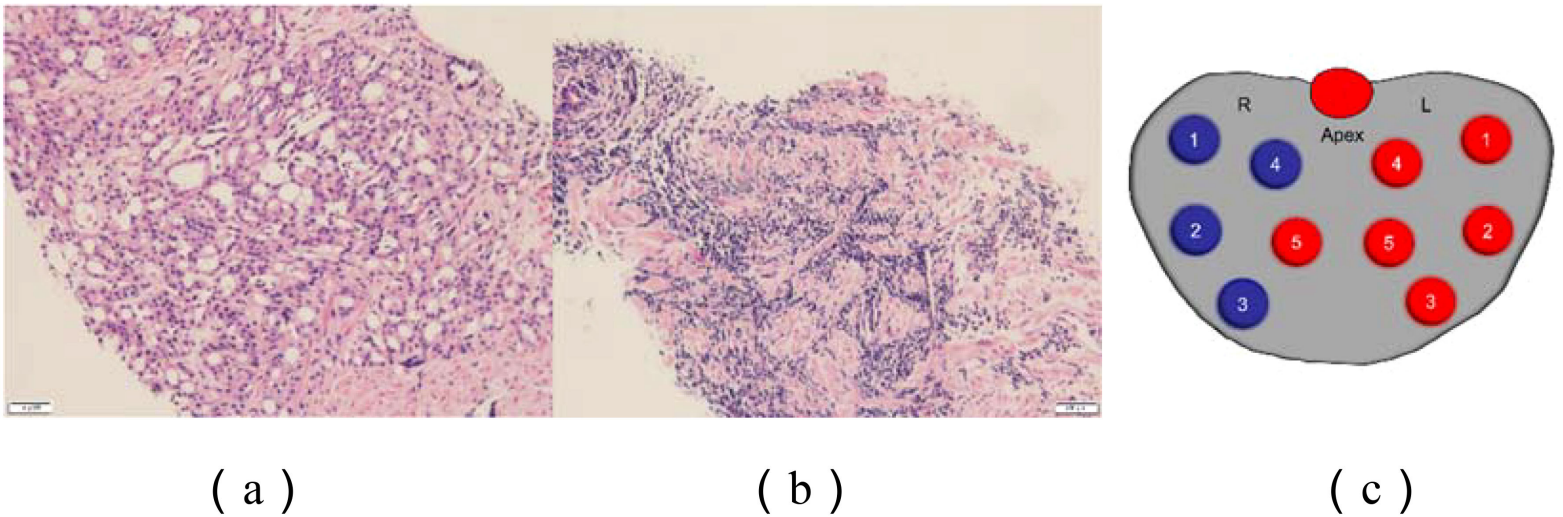
Prostate pathology. **(a)** Chronic lymphocytic leukemia; **(b)** Prostate acinar carcinoma;**(c)** Prostate puncture site: Blue: Prostate acinar carcinoma Red: Chronic lymphocytic leukemia. Right 1: Prostatic adenocarcinoma, Gleason score 4+4=8, grading group 4/5, tumor tissue ~100%. Right 2: Prostatic adenocarcinoma, Gleason score 4+4=8, grading group 4/5, tumor tissue ~70%. Right 3: Prostatic adenocarcinoma, Gleason score 4+4=8, grading group 4/5, nerve invasion, tumor tissue ~70%. Right 4: Prostatic adenocarcinoma, Gleason score 4+4=8, grading group 4/5, tumor tissue ~80%. Left 1-5, Right 5, Apical: Benign with lymphocytic infiltration, indicative of small lymphocytic lymphoma (SLL).

**Figure 3:**
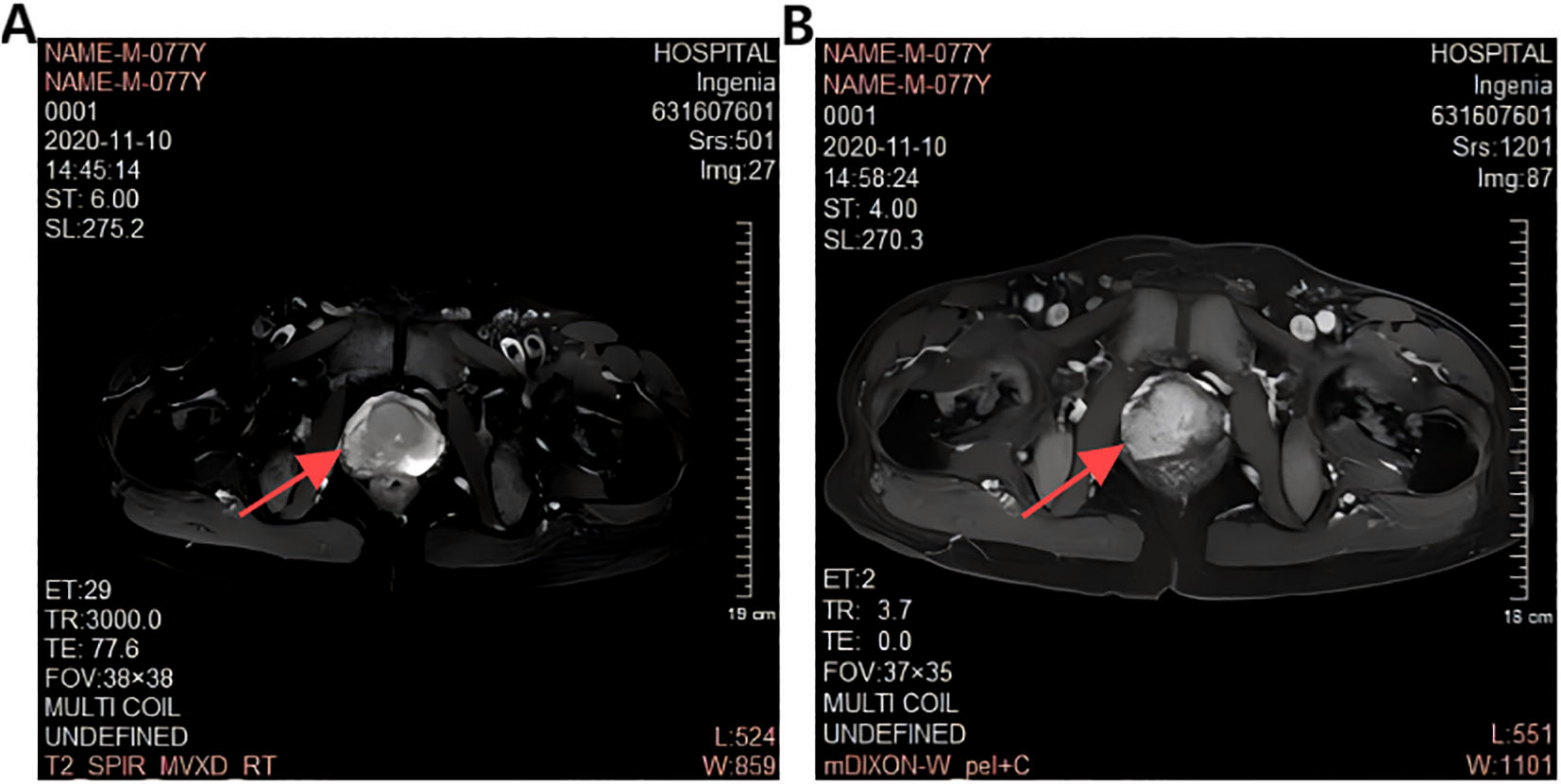
Prostate Magnetic Resonance Examination **(A)**: Unevenly decreased T2W1 signal in the peripheral band (red arrows). **(B)**: Enhanced DWI signal in the peripheral band (red arrows).

A 77-year-old male presented with urinary difficulty and a three-month history of nocturnal sweating. The complete blood count showed a markedly elevated white blood cell count (WBC: 39.35*10^9^/L), predominantly due to an increased proportion of lymphocytes. Urinalysis was negative for white blood cells. Tumor marker testing revealed an elevated PSA level of 75.26 U/mL (reference range: ≤4.0 U/mL). Ultrasound imaging indicated multiple enlarged lymph nodes in the neck, axilla, and groin, and prostate enlargement with abnormal signals in the right peripheral zone, suggesting a tumor or inflammation (**Figure 1**). The patient was diagnosed with malignant liver tumor and hepatitis B four years ago in an outside hospital, where the diagnosis of hepatocellular carcinoma was based on clinical and imaging assessment, and no liver puncture biopsy was performed, so the exact type of pathology could not be clarified. He had received interventional therapy and radiofrequency ablation and was now receiving conventional antiviral therapy for hepatitis B. Follow-up MRI showed resolution of the liver cancer lesions, with no signs of recurrence. A comprehensive review of the case revealed that the patient’s condition was multifaceted, with the potential for involvement of multiple systems and disciplines. To ensure a comprehensive diagnosis and precise treatment plan, an MDT consultation was initiated, with experts from relevant departments invited to conduct a comprehensive discussion. During this process, we meticulously reviewed the accuracy of the examination results with the teams of laboratory and imaging departments to ensure that the laboratory indicators and imaging manifestations were correct. We also invited the urology department to complete the prostate puncture and perfected the bone marrow puncture examination in the hematology department. The gastroenterologist’s evaluation confirmed the disappearance of the liver cancer lesion, with no evidence of recurrence. Furthermore, we conducted a thorough review of the pathological results from various puncture sites in conjunction with pathologists, thereby establishing a comprehensive foundation for subsequent diagnosis and treatment planning. The diagnostic process is delineated in **Figure 2**. Pathology of prostate biopsy showed that right cores 1, 2, 3 and 4 are consistent with prostate adenocarcinoma, left cores 1–5 and right core 5 together with the apex were consistent with small lymphocytic lymphoma (SLL) (**Figure 3**). Bone marrow cytology showed markedly active proliferation with 86% mature lymphocytes, suggesting a lymphoproliferative disorder in the bone marrow. Bone marrow biopsy pathology suggested small B-cell lymphoma involvement, consistent with chronic lymphocytic leukemia/small lymphocytic lymphoma (CLL/SLL). Immunophenotyping showed that 89% of the cells are lymphocytes expressing mainly HLA-DR, CD5, CD19, CD20, CD23, CD38, CD58, CD200, sLambda and BCL-2, but not FCM7, CD22, CD79b. Approximately 17% of the cells were CD5+CD19+CD23+. Myeloid proliferation was significantly suppressed, suggesting B-CLL/SLL-associated B-cell lymphoma/leukemia. Bone marrow chromosome analysis of 11 mesophilic cells, 7 of which were karyotyped with Y chromosome loss. TP53 gene mutation showed a missense mutation in exon 4. IGHV mutation detection indicated an IGHV mutation frequency of less than 2%.

The patient refused to undergo pelvic lymph node aspiration biopsy and PET-CT, and the final diagnosis was “chronic lymphocytic leukemia (CLL) with prostate involvement (Rai II stage, Binet B stage, TP53 mutation, IGHV unmutated, very high risk of CLL-IPI), prostate follicular adenocarcinoma (cT2bNxM0), and hepatocellular carcinoma after treatment. The patient exhibited a progressive increase in white blood cells, with a maximum white blood cell count of 174.15*10^9^/L and a lymphocyte count of 158.5*10^9^/L. In order to manage the disease, the patient commenced targeted therapy with ibrutinib (420 mg/day) on 19 December 2020. Due to the patient’s advanced age and their own refusal to undergo surgical treatment for prostate cancer, endocrine therapy for prostate cancer was initiated on 15 January 2021, with a regimen of goserelin (3.6 mg every 4 weeks) combined with bicalutamide (50 mg once a day). Following the treatment, a significant decrease was observed in peripheral blood leukocyte count, lymphocyte count, and PSA levels. As of January 14, 2022, the patient’s blood routine test results indicated a leukocyte count of: 5.34*10^9^/L and lymphocytes 1.85*10^9^/L. On 22 April 2021, the PSA count decreased to within normal parameters, and this remained within the normal range during subsequent follow-up.

On 27 January 2022, the patient underwent a further lymph node biopsy due to an axillary swelling, and was diagnosed with “diffuse large B-cell lymphoma (Richter’s transformation in CLL)”. This was considered to represent disease progression. Due to the patient’s advanced age, poor physical health, and refusal of immunochemotherapy, ibrutinib + rituximab immunotargeted therapy was selected without regular evaluation of the efficacy of the treatment. The patient died of an intracranial hemorrhage after a fall on 25 July 2022.

## Discussion

Multiple primary prostate malignant tumors are extremely rare, and even less common is the occurrence of adenocarcinoma of the prostate in conjunction with chronic lymphocytic leukemia (CLL), particularly in cases where each of the two cancers affects different areas of the prostate. The most common sites of involvement for CLL are the lymph nodes, the bone marrow, and the peripheral blood (7). The majority of documented cases in the extant literature pertain to prostate cancer in conjunction with chronic lymphocytic leukemia in other sites (devoid of prostate involvement), with the preponderance arising subsequent to the identification or management of chronic lymphocytic leukemia (8). There has only been one documented case of concurrent prostate cancer and CLL within the prostate. The patient, who presented with hematuria and urinary obstruction, had a prostate biopsy that initially did not reveal malignant cells but was later diagnosed with CLL and prostate adenocarcinoma following a prostatectomy, and the patient died shortly after diagnosis (9). The early diagnosis of multiple primary malignant tumors is challenging, and treatment decisions are complex, requiring a multidisciplinary team (MDT) approach involving hematology, urology, radiology, and pathology to develop an individualized management strategy. The patient exhibited a medical history marked by hepatocellular carcinoma, and upon this admission, a markedly elevated prostate-specific antigen (PSA) level was observed, accompanied by elevated white blood cell and lymphocyte counts. This finding indicated the presence of a multisystem abnormality. Following a multidisciplinary team (MDT) discussion, it was initially determined that the prostate lesion in question might contain both solid and hematopoietic tumor components, and the final diagnosis was prostate vesicular adenocarcinoma combined with chronic lymphocytic leukemia (CLL) prostate infiltration.

From a mechanistic perspective, the initiation and progression of tumors are regarded as complex, multistep and multifactorial biological processes. These processes are fundamentally driven by the continuous accumulation and evolution of genomically unstable clonal cell populations (10–12). Apparently, various intrinsic and extrinsic stresses, including hypoxia, nutritional deprivation, chemotherapy or radiotherapy, contribute to the survival and proliferative advantage of tumor cells carrying specific genomic aberrations, and then these cells gradually expand to dominate the tumor landscape (13). This process is known as cancer genome evolution and is the basis of both tumor heterogeneity and the development of multiple primary malignant tumors (14). In this particular context, mutations in TP53, a classical tumor suppressor gene that plays a key role in maintaining genomic stability, regulating the cell cycle and promoting apoptosis, can lead to the inactivation of downstream signaling pathways and impair the DNA damage response (15). Furthermore, certain gain-of-function mutations may actively promote tumor cell proliferation, invasiveness, and immune evasion, thereby accelerating clonal expansion and malignant progression (16). In prostate cancer, TP53 mutations have been demonstrated to be closely associated with high-grade differentiation, disease progression, and therapeutic resistance, while in chronic lymphocytic leukemia (CLL), TP53 aberrations are well-established molecular markers of poor prognosis (17, 18). In the present case, the detection of a TP53 mutation suggests that it may serve as a central driver of dual malignant clonal evolution. In the case under consideration, the presence of a TP53 mutation may indicate its role as a central driver of dual malignant clonal evolution. While there is currently no direct evidence linking the patient’s prior treatment for hepatocellular carcinoma, which included interventional embolization and radiofrequency ablation, to the subsequent development of prostate cancer and chronic lymphocytic leukemia (CLL), it is conceivable that these therapeutic interventions may have altered the internal microenvironment or exerted selection pressures that facilitated the expansion of TP53-mutant clones.

The management of multiple primary malignant tumors presents a significant challenge. Some reports have discussed the development of an appropriate treatment strategy that covers both cancers without increasing toxicity or drug interactions and without negatively impacting the patient’s overall prognosis (19). It is notable that there is currently no established set of guidelines for the treatment of MPMT of the prostate (two tumors present simultaneously in the prostate). The patient suffered from both prostate acinar adenocarcinoma and SLL/CLL involving the prostate. Choosing the treatment option with the greatest benefit was crucial, and it was challenging to decide whether to prioritize surgical interventions or medical management. Guidelines for the treatment of prostate adenocarcinoma include radical prostatectomy with pelvic lymph node dissection, external beam radiotherapy (EBRT) plus androgen deprivation therapy (ADT), or ADT alone (20, 21).However, as the patient is elderly with poor tolerance to surgery and radiation, and the lymphoma cannot be treated at the same time as surgery, this can lead to progression of the lymphoma. For the treatment of SLL/CLL, guidelines and research recommend prioritizing Bruton’s tyrosine kinase (BTK) inhibitors for TP53-positive chronic lymphocytic leukemia (7, 22). However, these drugs carry risks such as atrial fibrillation and bleeding, making them unsuitable for concurrent surgical treatment. Studies suggest that stopping treatment for more than a week after disease stabilization may reduce efficacy. By combining the Chinese Society of Clinical Oncology (CSCO), National Comprehensive Cancer Network (NCCN), and other international guidelines, as well as considering the patient’s age, cardiopulmonary, hepatic, and renal functions, disease progression speed, drug interactions, surgical tolerance, and the patient’s treatment preferences, we proposed a treatment plan for this case of multiple primary malignant tumors of the prostate (prostate adenocarcinoma combined with prostate lymphoma).

In this case, the patient opted for a combination of BTK inhibitors and medical castration (goserelin 3.6 mg q4w, bicalutamide 50 mg once a day). Regular monitoring has shown significant reductions in white blood cells, lymphocytes, and Prostate-Specific Antigen (PSA) levels, indicating effective treatment.

In conclusion, this case represents an extremely rare instance of prostate adenocarcinoma combined with CLL prostate infiltration with multiple primary malignant tumors. The pathogenesis of this condition remains unclear and may be related to TP53 mutation and clonal evolution, suggesting the need to focus on the molecular basis of multi-tumor co-occurrence. There is currently a paucity of guidelines for the treatment of prostate MPMT, and individualized regimens need to be developed taking into account the patient’s systemic condition, disease progression, and multidisciplinary evaluation. In this particular instance, the combination of a BTK inhibitor with pharmacologic debulking therapy was employed, yielding favorable outcomes. This case represents a rare clinical phenomenon, underscoring the necessity for enhanced identification and comprehensive management of multiple primary malignant tumors in clinical practice. While this case is unique, its rarity offers significant insights into the pathogenesis and therapeutic strategies of prostate MPMT.

## Data Availability

The original contributions presented in the study are included in the article/supplementary material. Further inquiries can be directed to the corresponding authors.
